# Floral Closure in Lesser Celandine (*Ficaria verna*) Protects Anthers from Pollen Flushing and Preserves Pollen Viability

**DOI:** 10.3390/plants14101437

**Published:** 2025-05-11

**Authors:** Pavol Prokop, Zuzana Provazník, Kristián Tučník

**Affiliations:** 1 Department of Environmental Ecology and Landscape Management, Faculty of Natural Sciences, Comenius University, Ilkovičova 6, 842 15 Bratislava, Slovakia; 2 Institute of Zoology, Slovak Academy of Sciences, Dúbravská cesta 9, 845 06 Bratislava, Slovakia; zuzana.provaznik@uniba.sk (Z.P.); tucnik1@uniba.sk (K.T.)

**Keywords:** flower evolution, floral movements, pollen protection, rain avoidance

## Abstract

Flower closure is a widespread yet understudied trait that may serve multiple functions in the success of plant reproduction. In this study, we investigated the role of flower closure in protecting pollen from rain-induced loss in lesser celandine (*Ficaria verna* Huds., 1762), an early-flowering species vulnerable to spring rains. Through simulated and natural rain experiments, we found that the flowers that were prevented from closing retained significantly fewer pollen grains compared to the control flowers. This demonstrates that flower closure effectively protects pollen from rain-induced flushing, thus enhancing reproductive success. Furthermore, flowers that were prevented from closing had fewer viable pollen grains than control flowers. We propose that the evolution of petal movement in *F. verna* was primarily driven by pressures favoring petal movement that protected pollen, with secondary contributions from herbivore avoidance. Flowers are unable to discriminate between low luminosity caused by cloudy weather and night, thus responding to both. Future studies should explore the relative importance of primary and secondary evolutionary drivers of flower closure across species, particularly in early-flowering plants facing complex environmental challenges.

## 1. Introduction

The primary function of flowers is to facilitate reproduction through pollination and fertilization [1]. Flowers achieve this through a combination of morphological traits (e.g., color, size, and shape) [2,3] and non-morphological traits, such as floral movements (e.g., flower opening and closure) [4]. While both traits contribute to reproductive success, the role of flower movements in enhancing fitness remains poorly understood.

Flower closure can protect reproductive organs from biotic (e.g., predation and regulation of pollinator visitation) and abiotic factors (e.g., temperature fluctuations, rain, and wind) [5,6,7]. By maintaining optimal microenvironmental conditions, intact flowers enhance pollen viability [8,9]. Conversely, flowers prevented from closing exhibit reduced reproductive success and increased vulnerability to predation [10,11,12,13,14].

Rain, while essential for plant growth, can negatively impact pollen availability by physically dislodging pollen grains from anthers [15,16,17]. Evidence from *Tulipa iliensis*, *Gentiana algida*, and *Merremia umbellata* suggests that flower closure reduces pollen loss under both simulated and natural rain conditions [9,10,11]. However, studies directly linking flower closure to pollen retention under natural conditions remain limited, particularly in early-flowering species.

Here, we investigate the pollen protection hypothesis in the lesser celandine (*Ficaria verna*), an early-flowering terrestrial plant. *Ficaria verna* exhibits repeated flower opening and closing, a behavior thought to protect against herbivory [12]. However, like other multifunctional structures shaped by diverse selective pressures (e.g., trichomes and cactus spines) [18,19], flower closure in *F. verna* may also safeguard pollen from rain-induced loss and/or maintain pollen viability. We hypothesized that repeated flower closure in *F. verna* protects pollen from rain, thereby enhancing reproductive success. Furthermore, we hypothesized that pollen viability is lower in flowers that are prevented from closing than intact flowers.

## 2. Results

### 2.1. Simulated Rain Experiment

**Figure 1:**
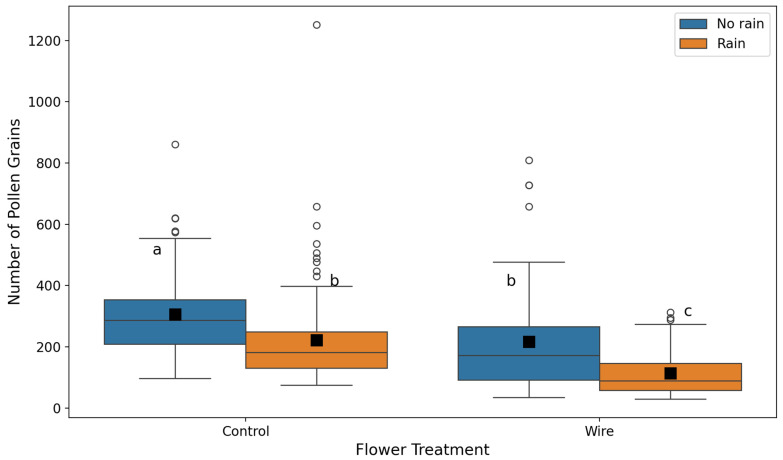
Differences in the number of pollen grains between control and wire-treated flowers with respect to the presence of simulated rain. The box shows the interquartile range (IQR), the horizontal line is the median, black squares show the mean, whiskers extend to 1.5 × IQR beyond the 1st and 3rd quartiles, and points beyond whiskers represent outliers. Different letters denote significant differences based on post hoc tests (a vs. b, a vs. c, b vs. c, *p* < 0.001).

The mean number of pollen grains in flowers exposed to simulated rain was significantly lower than in those not exposed to simulated rain (GLMM, estimate = −0.498, χ^2^ = 3394, df = 1, *p* < 0.001, Figure 1). The mean number of pollen grains in untreated flowers exposed to simulated rain was significantly lower than in flowers prevented from closing (GLMM, estimate = 5.27, χ^2^ = 3247, df = 1, *p* < 0.001, Figure 1). The interaction term between the variables was significant (GLMM, estimate = −0.30, χ^2^ = 309, df = 1, *p* < 0.001, Figure 1). This suggests that control flowers that were prevented from simulated rain showed a similar number of pollen grains as those that were prevented from both simulated rain and closure (Bonferroni post hoc test, *p* = 1.0). All other differences between the groups were statistically significant (Bonferroni post hoc tests, all *p* < 0.001).

### 2.2. Natural Rain

**Figure 2:**
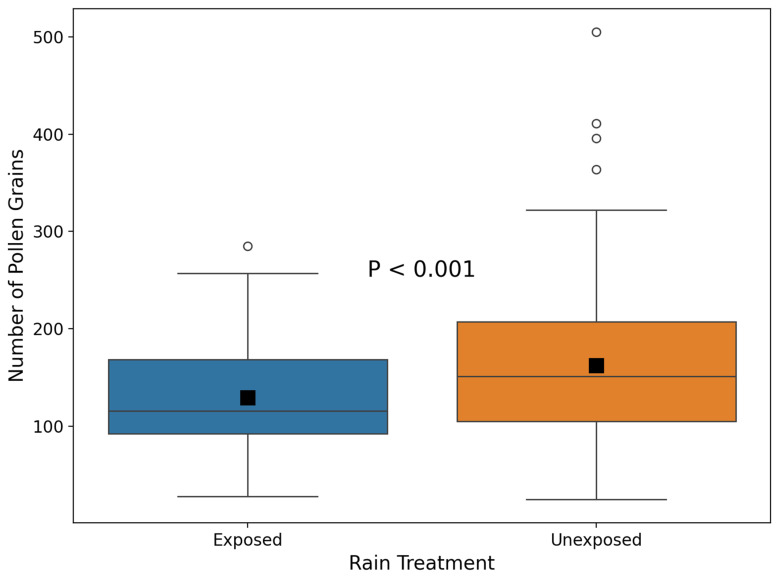
Differences in the number of pollen grains between wire-treated flowers exposed and unexposed to natural rain. For designations, see Figure 1.

A previous experiment showed that flower closure protects anthers from pollen flushing by simulated rain. Here, we examined whether flowers that were prevented from closing lost pollen grains as a result of rain. The mean number of pollen grains in flowers prevented from closing, exposed to natural rain, was significantly lower than in flowers prevented from closing, unexposed to natural rain (GLM, estimate = 0.23, χ^2^ = 349, df = 1, *p* < 0.001, Figure 2).

### 2.3. Pollen Viability

**Figure 3:**
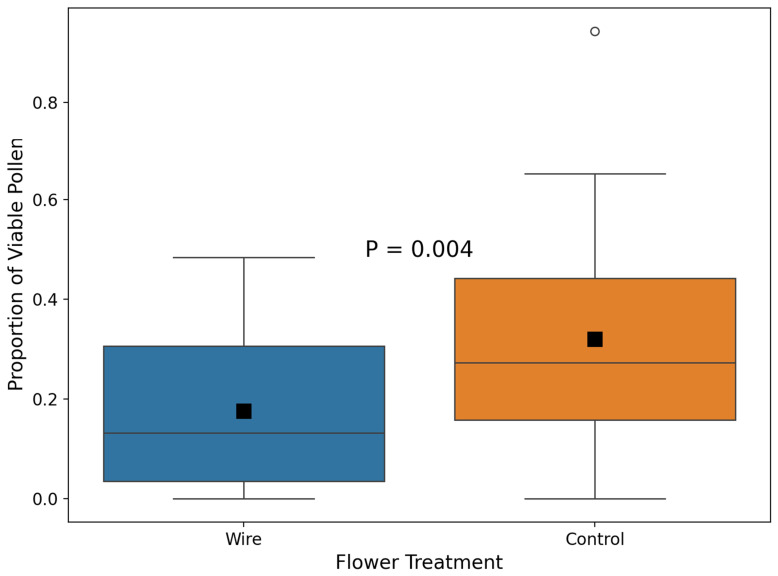
Differences in the proportion of viable pollen grains between wire-treated flowers and control flowers when both are exposed to natural conditions in the field. For designations, see Figure 1.

The proportion of viable pollen grains ranged from 0.00 to 0.48 in wire-treated flowers and from 0 to 0.94 in control flowers. The difference between groups was statistically significant (Brunner–Munzel test, W = −3.03, df = 58, *p* = 0.004, Figure 3). Wire-treated flowers contained samples with zero viable pollen grains at a similar frequency (5 out of 30) as control flowers (1 out of 30) (Fisher’s exact test, *p* = 0.20). Control flowers allowed to close their petals had a greater proportion of viable pollen grains than experimentally treated flowers prevented from closing.

## 3. Discussion

Our results demonstrate that flower closure in lesser celandine significantly reduces pollen loss under both simulated and natural rain conditions. Specifically, flowers that were prevented from closing lost more pollen when exposed to rain, providing strong evidence that flower closure serves as a protective mechanism against rain-induced pollen flushing. Our results agree with previous studies on *Merremia umbellata* [9], *Gentiana algida* [10], and *Tulipa iliensis* [11], which also found that flower closure mitigates pollen loss under rainy conditions. Furthermore, flowers prevented from closing showed significantly lower pollen viability, supporting the idea that flower closure has multiple functions, which are shaped by evolutionary pressures.

By protecting pollen from rain, flower closure probably enhances reproductive success in *F. verna*, particularly during early spring when rain events are frequent. This is critical for early-flowering species, which depend on efficient pollen transfer within a limited flowering period. Additionally, early-flowering plants bloom when trees are leafless, which increases their exposure to unfavorable environmental conditions such as rain and wind. As a result, protective mechanisms against rain are expected to play a critical role in their reproductive success [20]. These mechanisms are not perfect, as the anthers were consistently wet after rain (P. Prokop, pers. obs.). Like many other insect-pollinated species, the pollinators of *F. verna*—mainly flies, Hymenoptera, and beetles [21]—are typically active only during favorable weather conditions, such as periods of sunshine and dry air. By preventing pollen damage or loss during adverse weather, flower closure helps ensure that a viable supply of pollen remains available when conditions improve and pollinators become active. It is also possible that flower closure protects the nectar from being diluted by rain [22], maintaining its attractiveness to pollinators. Our findings also support the idea that flower closure serves multiple functions, including protection against herbivory [12] and pollen loss. This multifunctionality reflects the diverse selective pressures shaping floral traits, similar to other adaptive traits such as trichomes and spines [18,19].

**Figure 4:**
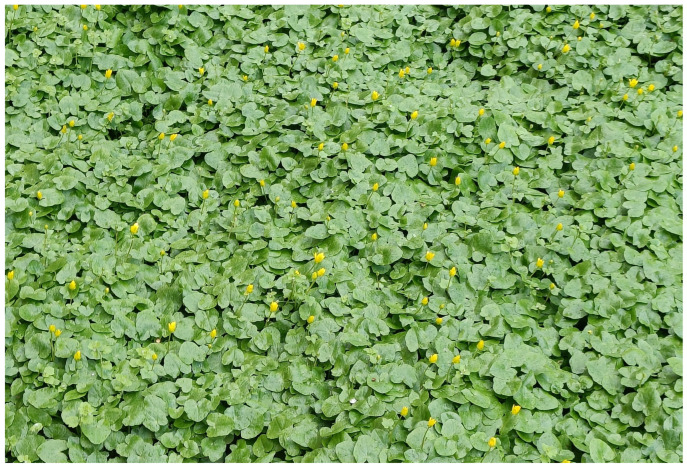
Almost uniformly closed flowers of lesser celandine during afternoon rainfall.

The evolution of flower closure in lesser celandine may be driven by both biotic (e.g., herbivory) [12] and abiotic (e.g., rain, this study) challenges. Because flower closure does not show apparent variability (flowers may slightly differ in the timing of closure but not in whether they close or not; P. Prokop, pers. obs.), we propose that the protection of anthers against pollen loss (and potentially pollen viability) is the primary driver of the evolution of petal movement. The coincidence of flower closure with herbivore activity [12] could further, although not primarily, contribute to the natural selection of flowers closing their petals in response to environmental cues associated with bad weather. Cloudy weather reduces luminosity, which could have been the initial selective force driving flower closure in this species (Figure 4). Because nights are also characterized by low luminosity, flowers may have been unable to distinguish between these two distinct environmental cues (i.e., cloudy weather and night), thus responding to both. This evolutionary mechanism can be explained by the smoke detector principle, which posits that adaptive traits often evolve to err on the side of caution [23]. In this case, the cost of failing to close during rain (e.g., pollen loss and reduced reproductive success) likely outweighs the cost of unnecessary closure during harmless conditions like nightfall. By evolving a generalized response to low luminosity, *F. verna* ensures protection against rain-induced pollen loss, even if it means occasionally closing when no rain is present. This conservative strategy is likely beneficial for the reproductive success of lesser celandine and may also influence the selection of diurnal pollinators.

Flowers experimentally prevented from closing (wire treatment) showed significantly fewer viable pollen grains than control flowers. Research showed that flower closure enhances pollen viability by preventing desiccation-sensitive pollen dispersal and protecting against contamination from molds and bacterial spores [24]. It also affects pollinator activity: delayed closure increases the likelihood of cross-pollination [25], whereas earlier closure promotes self-fertilization [11] and protects pollen from environmental stressors such as temperature and humidity fluctuations [4]. In *Gentiana straminea*, for instance, closure retains pollen on reproductive structures under cold conditions [26], while experimentally prevented closure reduces pollen viability, fruit set, and seed production while increasing seed abortion [9]. Similarly, interrupted petal closure in *Crocus discolor* lowers pollen viability, suggesting a protective role in early-flowering species facing harsh conditions [8]. Our research suggests that floral closure maintains optimal microclimatic conditions, protects pollen from environmental stressors, and enhances reproductive success in the lesser celandine.

There are several possible explanations for the reduced pollen viability observed in *F. verna* flowers that are prevented from closing. First, unprotected pollen can be damaged by excessive moisture from rain or dew. In some species, pollen grains are sensitive to hydration levels, and direct contact with water can cause premature swelling and bursting due to osmotic stress [27]. Additionally, persistent moisture may encourage fungal or bacterial growth on the pollen, increasing pollen mortality [28].

Second, pollen grains can be harmed by low temperatures [29]. *Ficaria verna* flowers naturally close overnight when temperatures drop. If kept open, the pollen is exposed directly to the cold, which can damage the cellular structures or inhibit the metabolic processes needed to maintain viability. It is possible that flower closure helps create a slightly warmer microclimate inside the flower, protecting the reproductive organs from chilling damage.

The multiple functions of flower closure in *F. verna* exemplify floral traits adapted to complex environmental pressures. However, more research is needed to explore how flower closure interacts with other factors, such as pollinator behavior and temperature fluctuations [26]. For instance, closed flowers at night could restrict unwanted pollinator access and influence stigma receptivity and pollen viability [9,11,24]. Comparative studies across multiple species [16] could also reveal whether flower closure is a widespread adaptation to rain-induced pollen loss.

## 4. Materials and Methods

### 4.1. Study Species

Lesser celandine (Ranunculaceae) is a perennial, early-flowering plant native to Europe, temperate Asia, and Northern Africa, typically blooming from March to April [30]. It reproduces both vegetatively through tubers and sexually via seeds. The plant produces yellow flowers measuring between 2 and 6 cm in diameter [31]. Its primary pollinators include hymenopterans, dipterans, and beetles [21]. The species is characterized by a consistent upward orientation of its flowers.

### 4.2. Study Area

Field experiments focusing on the number of pollen grains were conducted from 4 to 24 April 2021 near the Trnávka stream in Western Slovakia, close to the city of Trnava (48°39′ N, 17°57′ E). The second experiment, focused on pollen viability, was performed in the same locality from 4 to 8 April 2025. The daily and night temperatures during the field study varied between 4 and 17 °C and −2 and 6 °C, respectively. Flower buds were individually marked and numbered on the first day of opening with a ribbon attached to a peduncle to ensure that the flowers did not open previously. The selection of flowers and their assignment to treatments was random.

### 4.3. Procedure

**Figure 5:**
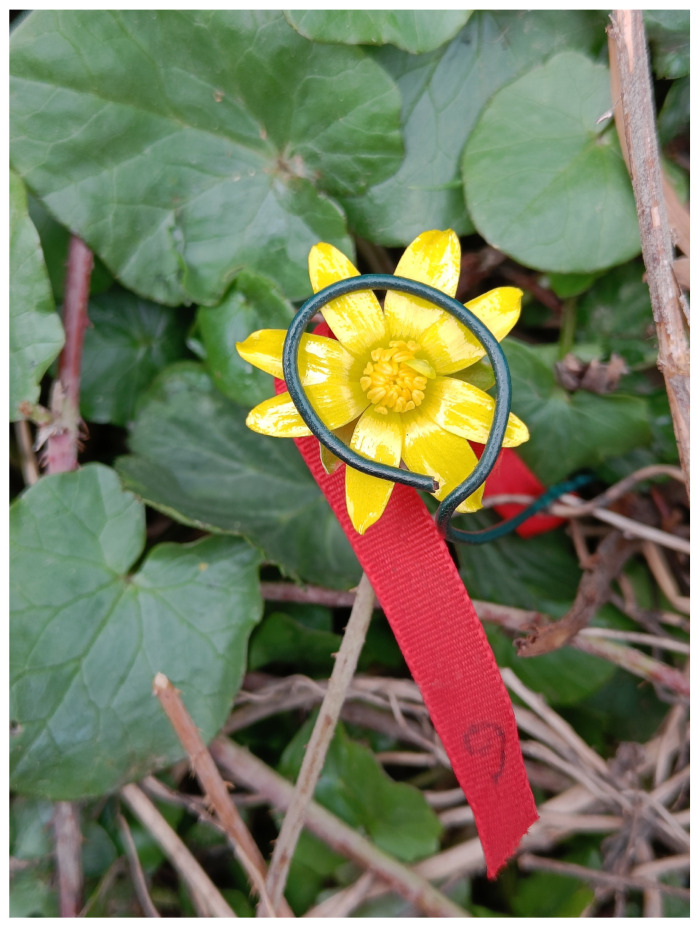
An experimental flower from the wire treatment.

In the experiment focusing on the number of pollen grains among flowers exposed to simulated rain, as well as in the experiment evaluating pollen viability, flowers were randomly assigned to two groups: flowers prevented from closing (wire treatment, Figure 5) and untreated flowers (control treatment). The flowers were treated with a wire that prevents flower closure [12]. Untreated flowers were left intact. Second, flowers were exposed or unexposed to rain (details below). Treating flowers with a wire took place between 16:00 and 18:00, when flowers were still open. Immediately after experiments with a number of pollen grains finished, we gently removed all flowers, placed them individually in plastic tubes, and separated all anthers from each flower before storing them in 70% ethanol. In experiments assessing pollen viability, flowers were individually placed in plastic tubes, transported to the laboratory, and immediately examined for their pollen viability as described below.

### 4.4. Simulated Rain Experiment

We conducted a simulated rain experiment in the field with flowers randomly divided into four treatments: flowers prevented from closing, exposed to simulated rain (*N* = 73); flowers prevented from closing, unexposed to simulated rain (*N* = 62); untreated flowers exposed to simulated rain (*N* = 94); and untreated flowers unexposed to simulated rain (*N* = 68). The ages of the flowers were not identical due to various environmental variables. For instance, marked flowers were closed during cloudy days and could not be prevented from closing. We used 2–4-day-old flowers, and their ages were controlled in statistical analyses (see below). Flowers exposed to simulated rain were irrigated with 3 L of water per square meter, equivalent to 3 mm of precipitation, a value consistent with typical rainfall patterns in the study area (P. Prokop, pers. obs.). This volume corresponds to the natural rainfall conditions described below. Water was applied using an irrigation apparatus equipped with a sprinkler head featuring 1 mm apertures. Watering always took place after 19:30, after sunset, when untreated flowers were close. Flowers were removed and placed individually into plastic tubes immediately after the experiment.

### 4.5. Natural Rain Experiment

In this experiment, we investigated whether natural rain flushes pollen from anthers from open flowers. Two-day-old flowers (*N* = 84) were treated with a wire before natural rain, which occurred at night on 12 April 2021. Night rainfall was estimated by the weather station roughly 6 km away from our study site to be 3.7 mm. Early in the morning, all flowers were individually placed in plastic tubes and transported to the laboratory. The same procedure was repeated on 26 April with *N* = 105 flowers, but with no rain. In other words, all flowers were prevented from closing, but approximately half of them were exposed to natural rain and half were not.

### 4.6. Pollen Count

Pollen grains were dislodged from the anthers using sonication in an ultrasound bath (Bandelin RK 31) for 10 min. The resulting solution was evaporated at 60 °C in the laboratory dryer (Memmert UF30). The dried pollen was then resuspended in 1 mL of a 70% ethanol/glycerol mixture (4:1 ratio) and subjected to another round of sonication. Using a micropipette, 10 μL of the solution was transferred in triplicate onto glass slides, covered with cover slips, and counted for all pollen grains. The total pollen count per flower was determined by multiplying the average number of pollen grains in the 10 μL samples by 100.

### 4.7. Pollen Viability

One-day-old flowers were treated with wire (*N* = 30), while another group of flowers (*N* = 30) was left intact. Flowers were collected after 5 days of exposure to the natural environment, during which rain occurred several times, with an estimated total rainfall of 25 mm. A pollen sample from each flower was carefully transferred to glass slides. A drop (30 μL) of solution containing 1% 3-(4,5-dimethyl-2-thiazolyl)-2,5-diphenyl-2H-tetrazolium bromide (MTT) in 5% sucrose (Sigma-Aldrich, St. Louis, MO, USA) was added and immediately covered with a coverslip [32]. After 20 min of incubation, pollen viability was observed under an optical microscope (LEICA DM 200 LED with 10 × 0.25 magnification). A pollen grain was considered viable if it turned dark. The proportion of dead pollen grains was calculated using a minimum sample size of *N* = 50 pollen grains.

### 4.8. Statistical Analyses

The number of pollen grains was treated as the dependent variable with a Poisson distribution, and flower treatment (untreated flowers and flowers prevented from closing) was defined as the categorical predictor in the Generalized Linear Model (GLM). The experiment with simulated rain was analyzed using a Generalized Linear Mixed Model (GLMM). Flower treatment (untreated flowers and flowers prevented from closing) and the exposure to simulated rain (flowers exposed or not) were defined as categorical predictors. Flower age was defined as the random effect, and the number of pollen grains was treated as the dependent variable with a Poisson distribution. Data on the proportion of viable pollen grains were not normally distributed (as determined by the Shapiro–Wilk test) and were compared using the Brunner–Munzel test. All tests were performed using the Jamovi software (https://www.jamovi.org/, accessed on 19 January 2025) [33].

## Data Availability

Data are available in the Supplementary Material.

## Supplementary Materials

The following supporting information can be downloaded at: https://www.mdpi.com/article/10.3390/plants14101437/s1.
